# CRISPR-Based Detection, Identification and Typing of Mycobacterium tuberculosis Complex Lineages

**DOI:** 10.1128/spectrum.02717-22

**Published:** 2023-01-05

**Authors:** A. Padane, Z. Harouna Hamidou, M. Drancourt, J. Saad

**Affiliations:** a Aix-Marseille-Université, IRD, MEPHI, IHU Méditerranée Infection, Marseille, France; b IHU Méditerranée Infection, Marseille, France; c Institut de Recherche en Santé, de Surveillance Épidémiologique et de Formation (IRESSEF), Dakar, Sénégal; d Laboratoire National de Référence des IST/VIH et de la Tuberculose, Niamey, Niger; Institut National de Santé Publique du Québec

**Keywords:** tuberculosis, clinical diagnosis, lineages, PCR, CRISPR-csm4, GeneXpert, *Mycobacterium tuberculosis* complex, Beijing lineage

## Abstract

The polymerase chain reaction (PCR)-based detection of Mycobacterium tuberculosis (M. tuberculosis) complex (MTC) in clinical samples is a first-line approach by which to diagnose tuberculosis in clinical microbiology laboratories. In this study, the genome-wide profiling of 3,156 mycobacterial genomes using Roary determined the CRISPR-csm4 gene as specific for MTB. Real time (RT)-PCR and the PCR-sequencing of CRISPR-csm4, tested on a collection of 20 MTC and 5 nontuberculous mycobacteria, confirmed the 20 MTC isolates, whereas the 5 nontuberculous isolates were not detected. Further, 65 of the leftover clinical samples, including 25 GeneXpert-positive and 40 GeneXpert-negative samples, that were used to evaluate the CRISPR-csm4-MTB assay in the clinical microbiology laboratory setting yielded expected results in every case, further allowing for the identification of the M. tuberculosis Beijing lineage. RT-PCR and the PCR-sequencing of CRISPR-csm4 could be implanted in the clinical microbiology laboratory to complement the currently used assays, with the potential of increasing the specification of the MTC pathogens responsible for tuberculosis.

**IMPORTANCE** The whole-genome sequence comparison of the Mycobacterium tuberculosis complex (MTC) genomic sequences that are available in the NCBI database identified a unique, specific gene to be used directly on clinical diagnostic samples to detect MTC against all species of mycobacteria and to differentiate between MTC species, lineages, and sublineages.

## INTRODUCTION

Out of 13 different species forming the Mycobacterium tuberculosis (M. tuberculosis) complex (MTC), only M. tuberculosis *sensu stricto*, Mycobacterium africanum (M. africanum), Mycobacterium bovis (M. bovis) as well as its derivative M. bovis bacille bilié Calmette-Guérin (BCG), and Mycobacterium canettii (M. canettii) (1) have been regularly detected as being responsible for tuberculosis (TB). This deadly infectious disease is still considered to be a worldwide health problem (2), and it is also of consideration to veterinary medicine (3). The identification of MTC isolates at the species level is of interest for the immediate medical diagnosis of the patient; for the tuberculosis treatment, as M. bovis and BCG are two species that are known to be pyrazinamide-resistant (4); and for source and contact tracing, as M. bovis is responsible for zoonotic tuberculosis (5) and BCG for care-associated tuberculosis (6). Today, real-time PCR (RT-PCR) and line probe tests based on the partial amplification of MTC DNA are commonly used to detect MTC mycobacteria directly in clinical samples. These techniques are now widely used for the routine diagnosis of TB, but they do not allow for the specific identification of different MTC species (7). Therefore, the identification of the M. tuberculosis *sensu stricto* lineage and sublineage is carried out through DNA sequence searches and whole-genome sequencing (WGS) using the cultural isolates (7). However, these efficient techniques require specific equipment that is not always available at the point of care (POC) laboratory, which delays the results by a few additional days or weeks (5).

In this study, WGS comparison of the MTC genomic sequences that are available in the NCBI database identified a unique specific gene that can be used directly on clinical diagnostic samples to detect MTC against all species of mycobacteria and to differentiate between MTC species and lineages.

## RESULTS

### Pangenome comparison analysis.

The pangenome of the 3,156 mycobacteria genomes used contained 264,486 genes. Based on the Roary output file for the presence or absence of genes, we detected 96 unique genes for MTC (Table S3). To confirm these unique genes, the Nucleotide Basic Local Alignment Search Tool (BLASTN) of these 96 genes against the nucleotide (nt) collection of the National Center for Biotechnology Information (NCBI) database, excluding the M. tuberculosis complex (taxid: 77643), showed no similar sequence in the NCBI (nt) database. As a result, these genes are unique in the MTC, and we investigated whether these genes exist in other species of mycobacteria. Next, we selected an annotated gene as CRISPR-csm4 from 96 unique genes to design specific primers with which to distinguish species and several lineages at the MTC. We have chosen to design two standard PCR systems and two RT-PCR systems.

### *In silico* validation, based on local clinical genomes.

A total of 145 genomic sequences for different genotypes of M. tuberculosis in the IHU Méditerranée Infection were used to search for the CRISPR-csm4 gene (Table S2). The alignment of these 145 clinical genomes indicated that all of the M. tuberculosis lineage Beijing had a 552 bp deletion fragment at the C-terminal end of CRISPR-csm4 (Fig. S1). In addition, two sequences (CSURQ1464 and CSURP9526) had a 474 bp deletion at the N-terminal end of the gene. We observed that M. tuberculosis and M. canettii had a common A-807 specific mutation against all other MTC species and that M. pinnipedii had a G-739 specific mutation. The *M. canettii* CRISPR-csm4 gene is heterogeneous among other sequences. Finally, it should be noted that no difference was observed between M. africanum 5 (type 1) and *M. afircanum* 6 (type 2) at the level of the CRISPR-csm4 gene.

### Design *in silico* of PCR systems.

Based on the CRISPR-csm4 gene alignment profile of 154 clinical samples with reference MTC genomic sequences, including 9 MTC references (Fig. S1), we used Primer-BLAST to design a specific primer. The first PCR system designed was ST1 (F-1 AGTCGTCCACGATTAGCTGC and R1-GTAATCGGGCCCCACATAGG), which detected all of the M. tuberculosis lineages, except for the two clinical isolates CSURQ1464 and CSURP9526. A second ST2 system, incorporating the PCR F2-GACGCTCACGACATCCCTAC and R2-GCGTAGCTGTAGACCGGATG with the same PCR parameter, differentiated M. tuberculosis, the M. tuberculosis Beijing lines, *M. canettii*, and *M. pinnipedii* against all species of the MTC and detected all of the M. tuberculosis lineages studied, including CSURQ1464 and CSURP9526, but not the Beijing lineage (Fig. S2). The combination of these two systems makes it possible to detect all of the lineages and sublineages of the MTC and to differentiate several members of the MTC, such as M. tuberculosis, the M. tuberculosis Beijing lineages of M. tuberculosis, *M. canettii*, and *M. pinnipedii* (Fig. S2).

On the other hand, two RT-PCR systems have been designed *in silico* (RT-S1 and RT-S2 systems) to detect all of the lineages and sublineages of the MTC. RT-ST1: F-1 GGTTTTCGGAGCGTTTAACCTT and R-1 TTTAGATGTCGTTCGTTTGC, TAMRA-CTCACGACATCCCTACCCAC; RT-ST2: F-2 ACCCTCTACTCTGCTTTG and R-2 TCTTCTTCGCCAGCTTCTTC, TAMRA-AGCTGCTTGACGAACTGCTT (Table 1). Using these two systems, MTC was detected using the RT-ST1 system, which showed all of the lineages of the MTC, without the Beijing strain, and, through the RT-ST2, all of the lineages of the MTC were detected, except for the two clinical isolates CSURQ1464 and CSURP9526 (Fig. S3).

**TABLE 1:** List and sequences of PCR primers used in this study

Method	Targeted organism	Targeted sequences	Primers	Pb	Hybridization temperature
RT-PCR	Mycobacterium tuberculosis complex	CRISPR-Csm4	F1-GGTTTTCGGAGCGTTTAACCTT R1-TTTAGATGTCGTTCGTTTGC TAMRA-CTCACGACATCCCTACCCAC	22 20 20	60° for 30 seconds
			F2-ACCCTCTACTCTGCTTTG R2-TCTTCTTCGCCAGCTTCTTC TAMRA-AGCTGCTTGACGAACTGCTT	18 20 20	
PCR Standard			F1-AGTCGTCCACGATTAGCTGC R1-GTAATCGGGCCCCACATAGG	20 20	
			F2-GACGCTCACGACATCCCTAC R2-GCGTAGCTGTAGACCGGATG	20 20	

### Laboratory system validation test.

The designed PCR systems were tested on cultured isolates belonging to the MTC: M. tuberculosis CSURQ2919 (sublineage 4.3.4.1), M. tuberculosis CSURP7746 (sublineage 1.1.1), M. tuberculosis CSURQ1206 (sublineage 2.2.1), M. tuberculosis CSURP9450 (sublineage 3.1.1), M. tuberculosis CSURP9458 (sublineage 1.1.2), M. tuberculosis CSURQ2261 (sublineage 1.2.2), M. tuberculosis CSURQ0987 (sublineage 4.8), M. tuberculosis CSURQ3429 (sublineage 4.8), M. tuberculosis CSURP9934 (sublineage 1.1), M. tuberculosis CSURP7756 (sublineage 3.1.1), M. bovis BCG, M. bovis CSURP7222, M. africanum CSURP9834, M. bovis CSURP9137, M. africanum CSURP7201, M. bovis CSURQ0209, *M. canettii* CSURQ3751, and *M. canettii* CSURQ3752. The following nontuberculous mycobacteria were used as controls: M. ulcerans, M. marinum, M. fortuitum, and M. intracellulare.

**FIG 1:**
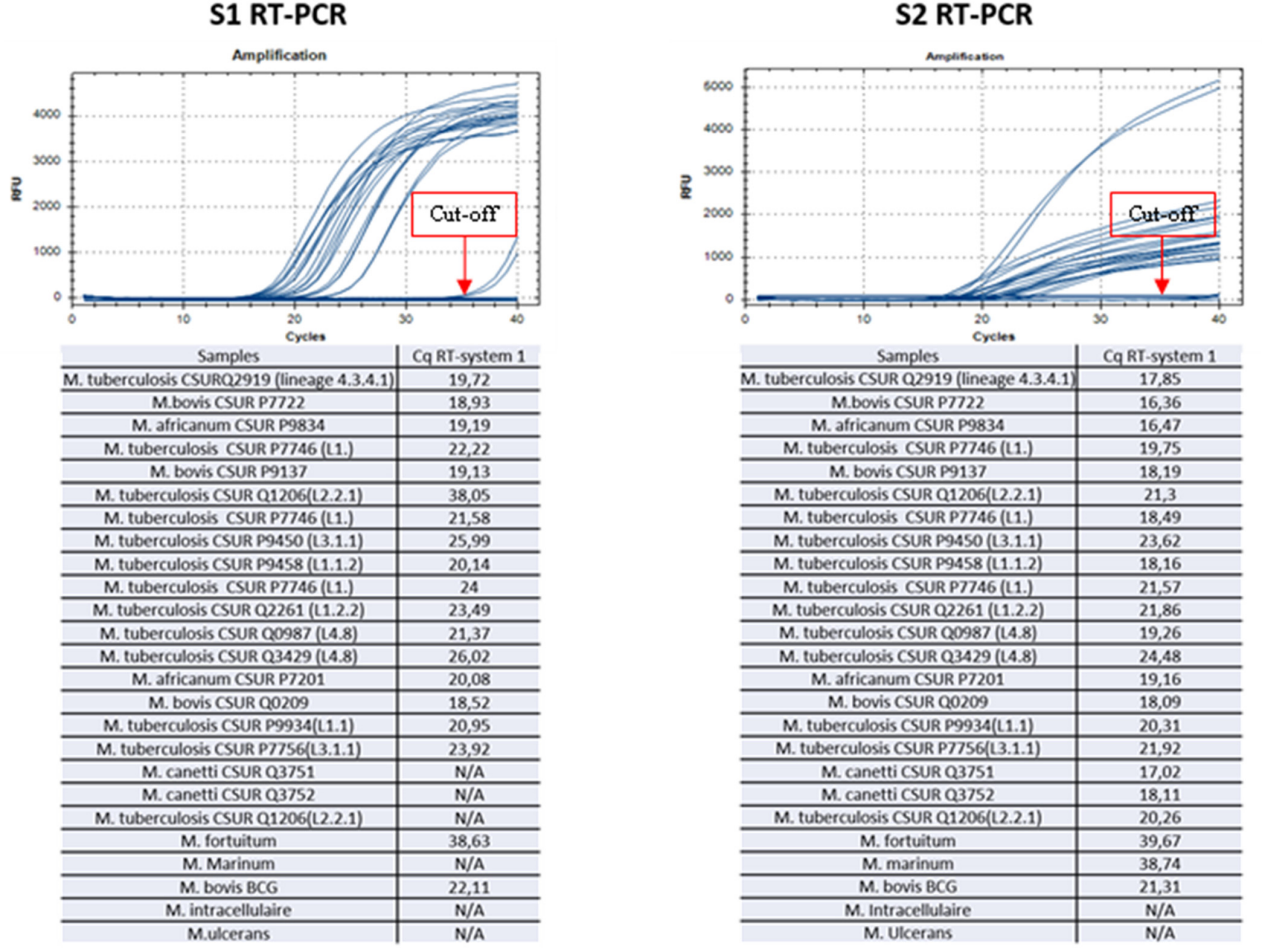
The results of the two RT-PCR systems (RT1 and RT2) on pure M. tuberculosis samples, with nontuberculosis Mycobacterium isolates as controls. This figure shows the CRISPR-Csm4 RT-PCR results and Ct values for the RT1 and RT2 systems on 20 Mycobacterium tuberculosis complex strains, 1 BCG strain, and 4 nontuberculous mycobacterial strains.

**FIG 2:**
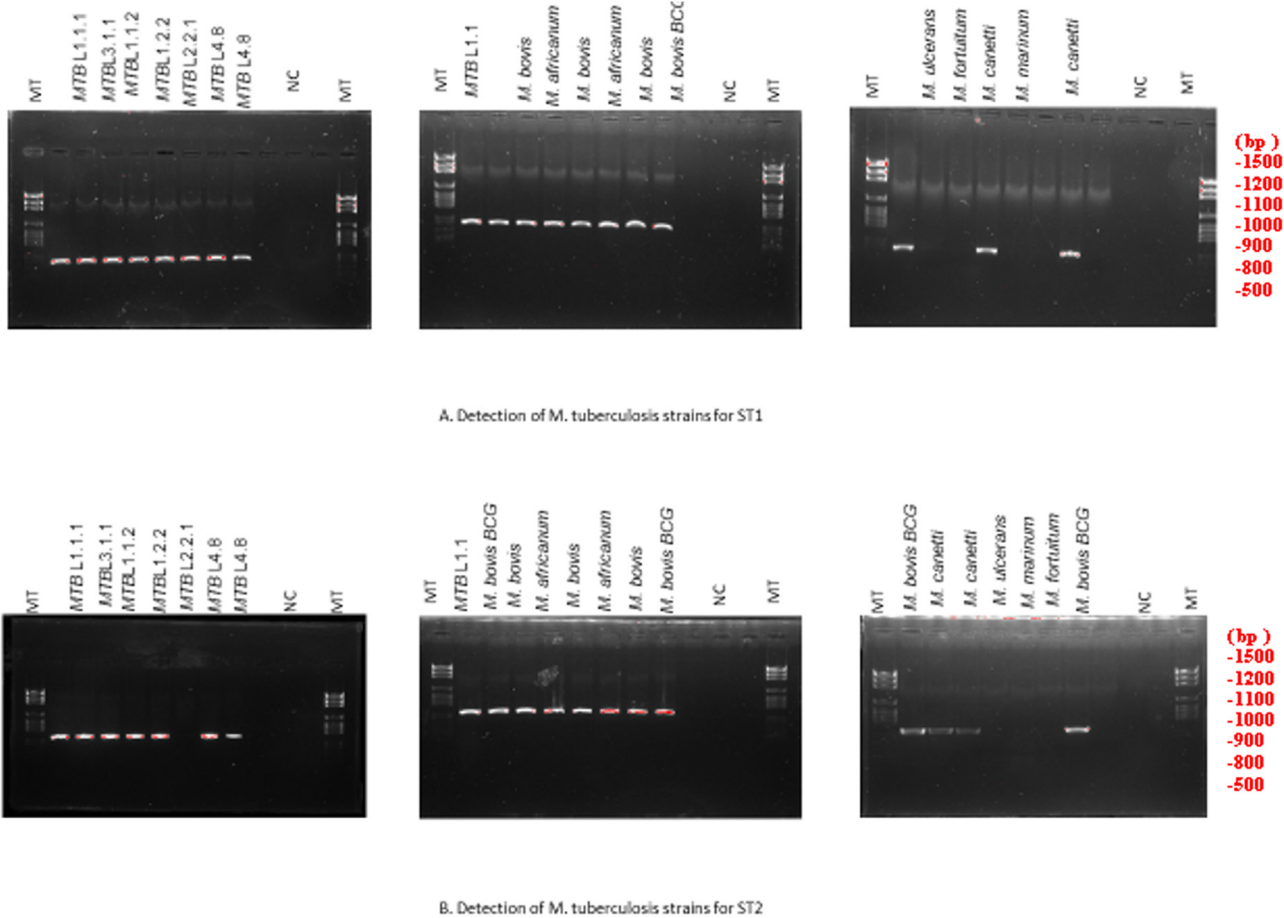
The results of the two standard PCR systems (ST1 and ST2) on pure M. tuberculosis samples, with nontuberculosis Mycobacterium as a control.

We observed that the RT-PCR systems RT-ST1 and RT-ST2 were negative with all of the control mycobacteria strains, whereas the RT-ST1 system was positive with all members of the MTC but negative with *M. canettii*, and the RT-ST1 system ST2 was positive with the entire MTC, including *M. canettii* (Fig. 1; Table 2). Standard PCR with two systems showed that 21 strains of MTC, including *M. canettii*, were detected by ST1 and ST2 and that four strains of nontuberculosis mycobacteria were not detected (Fig. 2). Additionally, 25 leftover sputum positive samples were positive in the RT-PCR and PCR sequencing. For 40 sputa, the negative controls were negative, using CRISPR-systems.

**TABLE 2:** Standard PCR pure strains results of ST1 and ST2 for the standard RT-PCR and PCR tests performed on 20 strains of the Mycobacterium tuberculosis complex, 1 strain of BCG, and 4 strains of nontuberculous mycobacteria

CSUR or strain identification	CRISPR standard PCR S1	CRISPR standard PCR S2	CRISPR standard PCR S1+ PCR S2
M. tuberculosis CSURQ2919 (L4.3.4.1)	+	+	+
M. bovis CSURP7222	+	+	+
M. africanum CSURP9834	+	+	+
M. tuberculosis CSURP7746 *(L1.1.1)*	+	+	+
M. bovis CSURP9137	+	+	+
M. tuberculosis CSURQ1206 (L2.2.1)	−	+	−/+
M. tuberculosis CSURQ2604 (L1.1.1)	+	+	+
M. tuberculosis CSURP9450 (L3.1.1)	+	+	+
M. tuberculosis CSURP9458 (L1.1.2)	+	+	+
M. tuberculosis CSURP2261 (L1.1.1)	+	+	+
M. bovis * P9472*	+	+	+
M. tuberculosis CSURQ0987 (L4.8)	+	+	+
M. tuberculosis CSURQ3429 (L4.8)	+	+	+
M. africanum CSUR P7201	+	+	+
M. bovis CSURQ0209	+	+	+
M. tuberculosis CSURP9934 (L1.1)	+	+	+
M. tuberculosis CSURP7756 (L3.1.1)	+	+	+
*M. canettii* CSURQ3751	−	+	+
*M. canettii* CSURQ3752	−	+	+
M. tuberculosis CSURP7228 (L2.2.1)	+	+	+
M. fortuitum	−	−	−
M. marinum	−	−	−
M. bovis BCG	+	+	+
M. intracellulare	−	−	−
M. ulcerans	−	−	−

**FIG 3:**
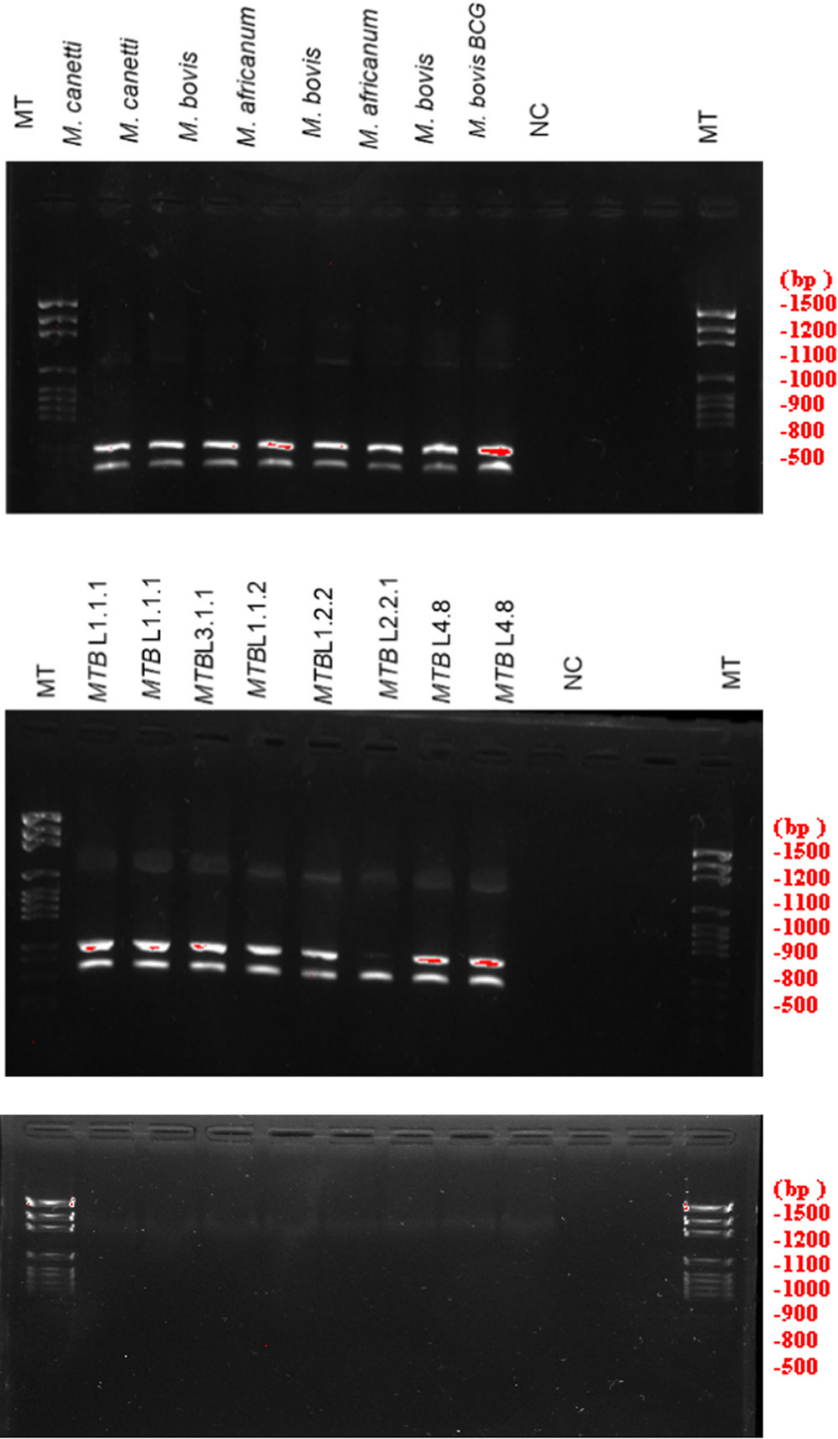
The results of the two standard PCR systems multiplex (ST1 and ST2) on pure M. tuberculosis samples with nontuberculosis Mycobacterium as controls. These figures show the standard CRISPR-Csm4 PCR results and Ct values for the S1 and S2 systems (Fig. 2) on 20 Mycobacterium tuberculosis complex strains, 1 BCG strain, and 4 nontuberculous mycobacterial strains. In addition, the results of these same strains are also shown by combining S1 and S2 using multiplex PCR (Fig. 3).

Indeed, our sequences showed the same lineage when we compared them with tuberculosis strains. Interestingly, it was possible to identify the Beijing strain by combining the two systems in standard PCR (Fig. 3). All of the positive samples in the standard PCR were sequenced, and the sequences were matched with laboratory results.

## DISCUSSION

In this study, a genome sequence-based comparison of M. tuberculosis was used to detect a specific MTC CRISPR-csm4 gene to be used as a target for PCR-based tests for the detection of the MTC and for the identification of genes in clinical diagnoses of M. tuberculosis. Among 96 genes of interest, we selected one hyperconserved gene, based on criteria such as monocopy and size. The repetitive CRISPR-csm4 gene, after an *in silico* specificity check using BLAST-n.p. (nucleotides and proteins) against the NCBI database, was detected in all of the sequences of the MTC genome that were available at the time of the study, including all 145 of the MTC genomes that were sequenced in our laboratory. The combination of these two systems can detect all of the lineages and sublineages of the MTC and can differentiate several members of the MTC, such as M. tuberculosis, the lineages of M. tuberculosis Beijing, *M. canettii*, and *M*. *pinnipedii*.

Furthermore, we showed that the CRISPR-csm4 sequence differentiated *in silico* between the principal species and lineages of the MTC. A previous study showed that residues 1 to 114 of CRISPR-csm4 were conserved across all 6 M. tuberculosis lineages but that residues 115 to 302 of Csm4, as well as Csm5, Csm6, Cas1, and Cas2, have been deleted in the lineage 2 (Beijing) strains (8), leading to the suggestion that the function of these proteins may be dispensable in M. tuberculosis.

Another study showed that the truncated Rv2820c in M. tuberculosis Beijing/W strains can improve the survival of M. tuberculosis in human macrophages, but it is unlikely that it will cause a different pattern of cytokine secretion by infected macrophages (9). This suggests that the truncated Rv2820c may be another Beijing/W specific virulence determinant (10). However, increased intracellular survival was not observed in the intact Rv2820c recombinant of the non-Beijing/W strains (9). In this study, we detected 2/145 clinical *M. tuberculosis* CSURQ1464 (Euro-American L4.2.2.1; genotype TUR) and CSURP9526 (Euro-American L4.3.3; genotype LAM) that had a large deletion in the CRISPR-csm4 gene (0 to 474 bp).

### Conclusion.

In this study, we successfully introduced a highly specific CRISPR-csm4 target gene with primers designated to detect M. tuberculosis complex species, based on a broad comparison of the genomes of the Mycobacterium genus. In addition, this gene of interest was profiled in 145 clinical samples of M. tuberculosis, including many known lineages and sublineages (Table S2). The data reported here indicated that the RT-PCR and PCR-sequencing of the CRISPR-csm4 gene, using the protocol developed here, could complement currently available molecular tools for the first line detection, identification, and genotyping of MTC mycobacteria in clinical samples.

## MATERIALS AND METHODS

### NGS data collection and pangenome comparison.

To select a single specific gene for the MTC, a total of 8,271 genomes were recovered from the NCBI assembly option until December 2020, using the keywords “Mycobacterium, *Mycolicibacterium*, *Mycolicibacter*, *Mycolicibacillus*, and *Mycobacteroides*”, and all of the M. tuberculosis genomes were removed, and a reference genome was selected for each species in the MTC to ultimately obtain 3,156 genomes (Table S1). The reference genomes selected in this study were M. orygis 112400015, M. africanum strain 25 (type 2), *M. orygis* strain NIRTAH144, *M. caprae* strain Allgaeu, M. bovis strain Danish 1331, *M. canettii* CIPT140010059, M. caprae MB2, M. tuberculosis H37Rv, M. tuberculosis 3-86Rv, M. mungi strain BM22813, M. microti strain 12, and *M. pinnipedii* strain ATCC BAA-688 (Table S1). The whole-genome profile of 3,156 genomes was performed using Roary (11), with a minimum 95% identity percentage for the protein and a 99% minimum percentage for a gene to be in the core, to detect unique MTC genes, relative to all mycobacteria. For all of the single genes detected, a selected monocopy gene, CRISPR-csm4, was used to design PCR and RT-PCR systems to identify and genotype MTC species of interest.

### *In silico* clinical validation of the unique gene and PCR designed tools.

To further validate the CRISPR-csm4 gene, we searched for the CRISPR-csm4 gene in the genome’s sequences of MTC isolates made in the IHU Méditerranée Infection, Marseille, France, between 2017 and 2019 (Table S2) (data from another unpublished study, NCBI project number: PRJEB39715). These 145 genomes were assembled using SPAdes version 3.13.1 (11) and were annotated using Prokka version 1.12 (12) to search for the gene in the annotated files. A PCR primer pair aimed to amplify the CRISPR-csm4 gene was designed using Primer-BLAST and Primer 3 (https://www.ncbi.nlm.nih.gov/tools/primer-blast/). In addition, CRISPR-csm4 has been investigated *in silico* for three type 1 M. africanum strains, including M. africanum ERR1215463, M. africanum ERR3170434, and M. africanum ERR1082124, using BLAST against the SRA database.

### Clinical studies.

A total of 25 MTC and nontuberculosis mycobacteria clinical isolates maintained in the “Collection de Souches de l’Unité des Rickettsies” (CSUR) within the NSB3 laboratory of the IHU Méditerranée Infection were cultured for 10 to 20 days on Middlebrook 7H10 agar medium supplemented with 10% oleic acid-albumin-dextrose-catalase (OADC) (Becton, Dickinson, Sparks, MD, USA) at 37°C in the same NSB3 laboratory. In addition, anonymized leftovers from samples that were routinely collected from patients who were diagnosed with pulmonary tuberculosis and extrapulmonary tuberculosis and who did not oppose to the preservation of such a clinical material were used in this study. No clinical material was sampled specifically for this study. Because no biographical data were associated with these anonymized leftover samples, and because the study therefore did not concern any individual, the use of these leftover samples did not require the advice of the Ethical Committee, as per the law in France. In detail, 25 leftovers of sputum samples that previously had been microscopically tested by Ziehl-Neelsen and had been found to be MTC positive by GeneXpert (Cepheid Inc., Sunnyvale, CA, USA) were assayed. The strains that were confirmed via culture were collected, and 40 sputa negative controls for validation isolates were used in this study. These samples followed the same confirmation procedures as did the positive samples. Then, the total DNA was extracted using a modified EZ1 protocol (Qiagen Tissue, Hilden, Qiagen, Germany) for all of the samples. Briefly, approximately 200 mg of colony or 50 μL of sample were mixed with 500 μL of G2 buffer and glass powder as well as 0.3 g of acid-washed beads of ≤106 μm (Sigma-Aldrich, Saint-Quentin Fallavier, France) in an Eppendorf tube. The mixture was vortexed for 45 s with FastPrep (MP Biomedical Europe, Illkirch, France), and it was then incubated at 100°C for 10 min. After 2 min of decantation, 180 μL of each sample were mixed with 20 μL of proteinase K (20 mg/mL) (Sigma-Aldrich) and were incubated at 56°C overnight. DNA extraction was carried out using an EZ1 device, following the manufacturer’s instructions (Qiagen). The DNAs were eluted under a 200 μL final volume and stored at −20°C.

### PCR-based detection and screening of the M. tuberculosis complex.

PCRs were performed using a Bio-Rad MyCycler CFX96 or a C1000 thermal cycler (Bio-Rad, Hercules, CA, USA). The PCR amplifications were carried out in a 50 μL volume containing 5 μL of the genomic DNA template, 25 μL of AmpliTaq Gold 360 Master Mix (Thermo Fisher Scientific), 1.5 μL of each primer (25 μM stock; forward primer and reverse primer), and 17 μL of deionized water. The PCR program for all of the designed systems included an initial denaturation at 95°C for 15 min, and this was followed by 35 cycles of 95°C for 30 s, 60°C for 30 s, and 72°C for 90 s. This was followed by a final extension at 72°C for 10 min. The PCR products were purified using a Millipore NucleoFast 96 PCR Kit, according to the manufacturer’s recommendations (Macherey-Nagel, Düren, Germany), and they were sequenced using a BigDye Terminator Cycle Sequencing Kit (Applied Biosystems) with an ABI automated sequencer (Applied Biosystems). The sequences were assembled using the UGENE version 34.0 software package (Technelysium Pty Ltd., Tewantin, Australia). Also, for the real-time PCR, the amplifications were carried out in a 20 μL volume containing 5 μL of the genomic DNA template, 10 μL of Mix Roche (2×) (Applied Biosystems), Master Mix (Thermo Fisher Scientific), 0.5 μL of each primer (20 μM stock; forward primer and reverse primer), 0.5 μL for the probe (5 μM stock), 3 μL of deionized water, and 0.5 μL of UDG. The amplifications were read with the program action UDG at 50°C for 2 min, with an initial denaturation at 95°C for 5 min, 39 cycles of denaturation at 95°C for 5 s, a hybridation at 60°C for 30 s, and a final elongation for 30 s.
