# Supplementary material for: CRISPR-Based Detection, Identification and Typing of Mycobacterium tuberculosis Complex Lineages
Source: Microbiol Spectr. 2023 Jan 5;11(1):e02717-22. doi: 10.1128/spectrum.02717-22 (PMC9927308; doi:10.1128/spectrum.02717-22)
Supplement: Supplemental file 1 — Supplemental material. Download spectrum.02717-22-s0001.pdf, PDF file, 1.0 MB [file spectrum.02717-22-s0001.pdf]

CRISPR-based detection, identification and typing of *Mycobacterium tuberculosis* complex lineages.

Abdou Padane[a,b,c], Zelika Harouna Hamidou[a,b,d], Michel Drancourt[a], Jamal Saad[a,b#]

[a] Aix-Marseille-Université, IRD, MEPHI, IHU Méditerranée Infection, Marseille, France

[b] IHU Méditerranée Infection, Marseille, France

[c] Institut de Recherche en Santé, de Surveillance Épidémiologique et de Formation (IRESSEF), Dakar, Sénégal

[d] Laboratoire National de Référence des IST/VIH et de la Tuberculose, Niamey, Niger

[#] Address correspondence to Dr Jamal SAAD Aix-Marseille University, MEPHI, IHU Méditerranée-Infection 19-21 Boulevard Jean Moulin, 13005 Marseille, France ; phone: +33658997312 ; e-mail : jsaad270@gmail.com

Supplementary tables:

Table S1. Recovered genomes list from NCBI assembly option database until December 2020 using the keywords “Mycobacterium, Mycolicibacterium, Mycolicibacter, Mycolicibacillus and Mycobacteroides,”

Table S2. Lineage and sublineage determined for 145 clinical *M. tuberculosis* isolates in IHU, Marseille, France. GenBank accession number of strains PRJEB39715.

Table S3. The 96 unique genes list for MTC detected in this study with nucleotide sequences.

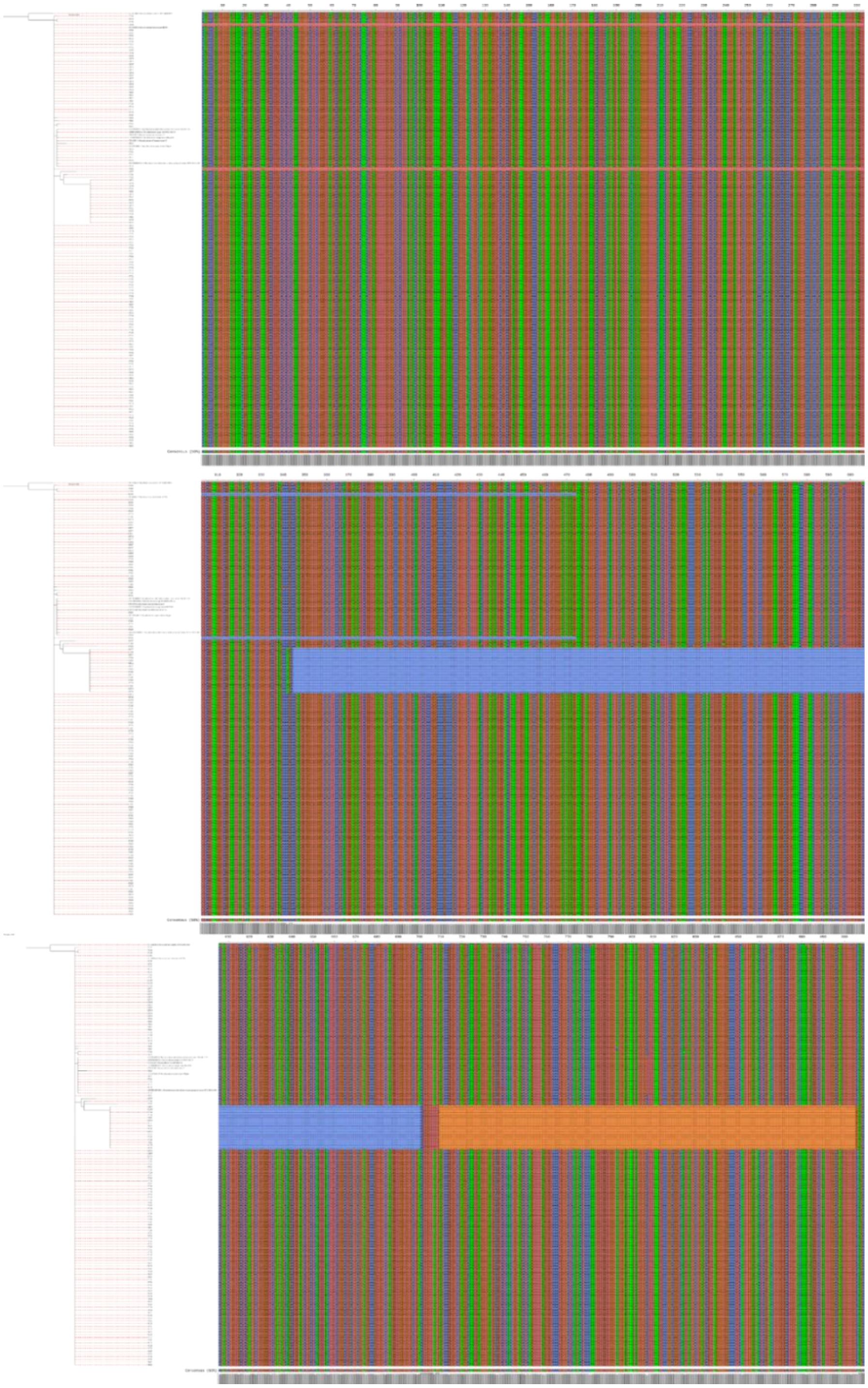

Figure S1. The alignment profile of 154 CRISPR-csm4 genes. Sequences were aligned using MAFFT 7.407_1 and Tree was generated using FastTree 2.1.11 on NGphylogeny website (https://ngphylogeny.fr/tools/tool/279/form)

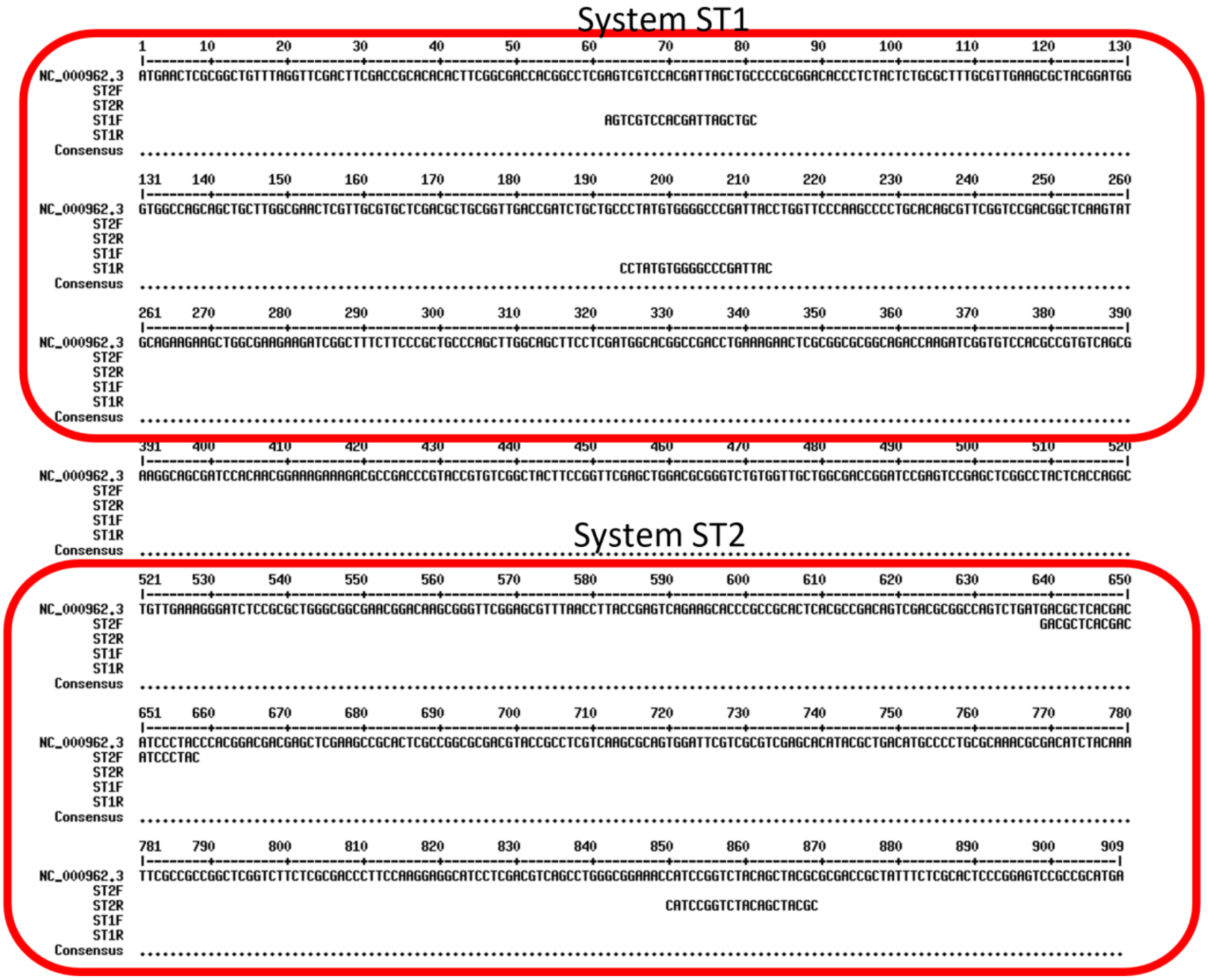

Figure S2. Alignment profile of the two PCR-standard systems with the *M. tuberculosis* H37Rv CRISPR-csm4 gene.

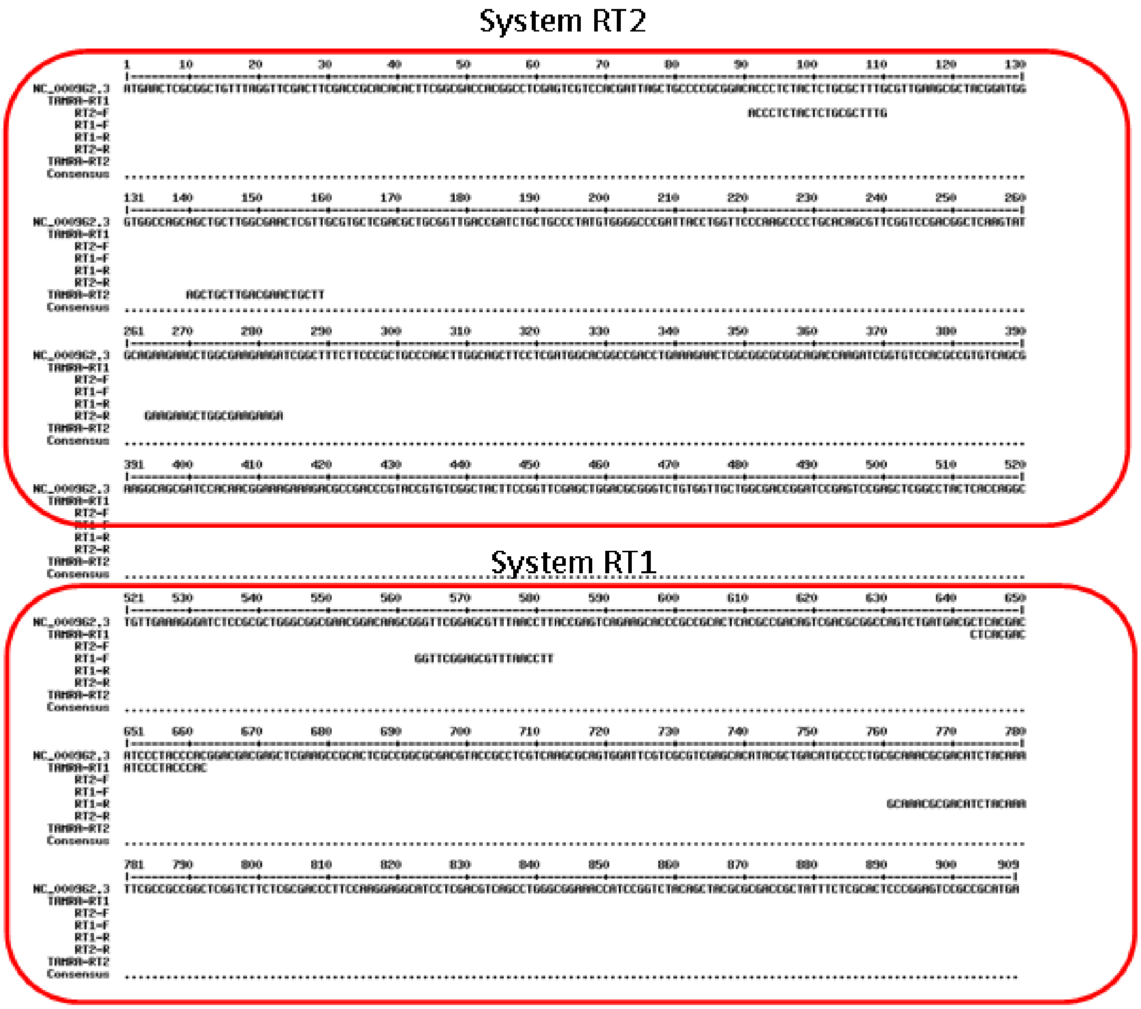

Figure S3. Alignment profile of the two RT-PCR systems with *M. tuberculosis* H37Rv CRISPR-csm4 gene**.**
